# Supplementary material for: Studies to Improve Perinatal Health through Diet and Lifestyle among South Asian Women Living in Canada: A Brief History and Future Research Directions
Source: Nutrients. 2021 Aug 24;13(9):2932. doi: 10.3390/nu13092932 (PMC8465246; doi:10.3390/nu13092932)
Supplement: Supplementary file 1 [file nutrients-13-02932-s001.zip › nutrients-1299202-supplementary.pdf]

Supplementary Material

Table S1. Laying the Groundwork for Culturally Tailored Interventions: Observations Cohort (START).

Study Name	Objective	Population	Outcome	Results	Implications
SouTh Asian biRth cohorT (START)	Investigate the environmental and genetic basis of adiposity among South Asian offspring recruited from highly divergent environments, namely, rural and urban India and urban Canada	1,012 Pregnant women and their offspring	Newborn and early childhood adiposity, and associated metabolic risk factors	Follow-up Ongoing; Children aged 5 years	-SA women have incidence of GDM of 33%<br>- newborns are low birthweight and have more adipose tissue<br>- Maternal diet influence of GDM risk and newborn birthweight

Table S2. Laying the Groundwork for Culturally Tailored Interventions: Qualitative Health Research.

Study Name	Objective	Population	Outcome	Results	Implications
START - Grandmothers study	Explore the perinatal health beliefs of South Asian grandmothers living in Canada	17 South Asian grandmothers	Grandmothers beliefs regarding optimal health behaviours for a woman before, during, and the first 6 weeks post pregnancy, and optimal behaviours for the family with the new baby in the first year of life	Grandmothers believe in the importance of healthy lifestyle habits around diet, activity and mental wellbeing at all times – preconception, pregnancy and post- partum	Provides an insight into the elder South Asian woman's cultural perceptions and beliefs around prenatal and postnatal health and wellbeing
START – HAPPY	To understand the South Asian mothers (from Ontario, Canada) views around Healthy Active Living, particularly in the form of exercise and diet supportive of blood glucose regulation.	15 South Asian mothers of childbearing age living in the Peel Region of Ontario, Canada	Barriers and facilitators of healthy active living for South Asian mothers of childbearing age	**Barriers**: Lack of knowledge, time and spousal/family pressures as well as lack of whole family engaging programs<br>**Facilitators**: knowledge, organization, access, commitment, technology, family activities, and relatable success stories	Understanding these barriers and facilitators will help us develop more culturally tailored and feasible interventions for promoting healthy active living among the South Asian families in the peel region
DESI-GDM Qualitative	To understand the cultural and contextual factors that influence the knowledge, attitudes, and practices of diet and physical activity of South Asian women of childbearing age and those who provide health care to this group.	10 pregnant or recently pregnant South Asian women<br><br>11 healthcare providers providing care to South Asian women during pregnancy	perceptions of health behaviours (diet and physical activity) during pregnancy in the South Asian community	**Women:**<br>Locus of control influences perceptions of overcoming barriers and challenges to healthy active living.<br>Support from family, friends, and healthcare providers is a highly valued.<br>**Healthcare providers:** (1) cultural awareness in caring for South Asian women during pregnancy; and (2) clinic management, logistics, and resources.<br>**Common theme** for both: importance of considering the cultural landscape in relation to how knowledge is obtained, shared, and valued.	A better understanding of these cultural underpinnings may support the development of interventions tailored for pregnant South Asian women and their healthcare providers.

Table S3. Laying the Groundwork for Culturally Tailored Interventions: Mixed Methods Research.

Study Name	Objective	Population	Outcome	Results	Implications
SMART START Evaluation Study (SS Evaluation Study	We aim to pilot test the SMART START intervention using qualitative and quantitative approaches.	20 Pregnant South Asian women, no previous diagnosis of diabetes, able to communicate (read and speak) in English<br><br>5 Family doctors: provides clinical care to a large volume of South Asian pregnant women, English speaking	The primary outcomes are feasibility and acceptability of the intervention (measured using a combination of mixed method tools: surveys and qualitative interviews).<br><br>The secondary outcomes are change in knowledge, attitudes, practices, and confidence (measured using a combination of mixed method tools: surveys and qualitative interviews).	In-Progress	Have a culturally tailored and evidence driven tool kit for pregnant mothers of South Asian origin and their care providers to help reduce their risk of developing gestational diabetes
START WATCH	Assess if structural supports can empower SA women as agents of change	1. South Asian women of childbearing age living in the Peel region and their community	1. Social Network Analysis (SNA) and exploratory case study to comprehensively explore and describe how SA women in the Peel Region promote healthy active living lifestyles within their families and social networks.<br>2. Using community readiness model to predict how willing and prepared a community is to address lifestyle change as it relates to NCD prevention	In Progress	SA women can act as agents of change who can reinforce and reiterate healthy active living messages (dietary, sleep, physical activity) within their social networks

Table S4. Laying the Groundwork for Culturally Tailored Interventions: Randomized Control Trial.

Study Name	Objective	Population	Outcome	Results	Implications
DESI-GDM RCT	Conduct a randomized controlled trial (RCT) of a culturally-tailored, personalized nutrition intervention to reduce gestational dysglycemia in pregnant South Asian women living in Peel region (Ontario).	190 South Asian pregnant mothers from the Peel region	The clinical outcomes of interest are the glycemic response to an oral glucose load, incident GDM, and infant birthweight.	Recruitment ongoing	A culturally tailored, personalized nutrition intervention delivered by a trained health coach to will result in reduced gestational dysglycemia, which may reduce the mother and child's future risk for diabetes and cardiovascular diseases
